# Use of penile shear wave elastosonography for the diagnosis of Peyronie’s Disease: a prospective case–control study

**DOI:** 10.1186/s12610-022-00164-w

**Published:** 2022-08-16

**Authors:** Francesco Trama, Ester Illiano, Fabrizio Iacono, Antonio Ruffo, Giovanni di Lauro, Achille Aveta, Felice Crocetto, Celeste Manfredi, Elisabetta Costantini

**Affiliations:** 1 Andrology and Urogynecology Clinic, Santa Maria Terni Hospital, University of Perugia, Perugia, Italy; 2 Department of General and Specialized Surgeries, Intensive Care and Pain Management, Renal Transplantation, University of Federico II, NephrologyNaples, Italy; 3 U.O. Urologia, Clinica Nostra Signora Di Lourdes, Massa di Somma, Naples, Italy; 4 UOC Urologia Santa Maria Delle Grazie, Pozzuoli, Italy; 5 Department of Woman, Child and General and Specialized Surgery, Urology Unit, University of Campania “Luigi Vanvitelli”, Naples, Italy

**Keywords:** Curvature, Elastography, Painful erection, Penile induration, Peyronie’s disease, Stiffness, Courbure, Elastographie, Erection douloureuse, Induration pénienne, Maladie de La Peyronie, Rigidité

## Abstract

**Background:**

To evaluate the stiffness of the tunica albuginea (TA), we used a new noninvasive diagnostic technique called shear wave elastography (SWE). We determined whether SWE values are correlated with the degree of penile curvature, the time of disease onset, and pain severity experienced by patients during erection. This study analyzed the elasticity of the TA of patients with Peyronie’s disease compared to that of the control group. We also analyzed any correlations between the stiffness of the cavernous bodies and the degree of curvature, time from diagnosis to curvature onset, and erectile pain severity. This was a prospective case–control study involving 100 men enrolled from September 2020 to August 2021. Participants were divided into group A (case group, *n* = 50), which included men with PD, with or without pain, and with penile curvature, or group B (control group, *n* = 50), which included healthy patients older than 18 years who visited the urology clinic for reasons other than PD. The medical history was collected for all patients who also underwent objective examination, B-mode ultrasound evaluation, and SWE. The International Index of Erectile Function (IIEF-15) visual analog scale (VAS) questionnaire was administered to all participants.

**Results:**

There were no significant between-group differences regarding age, weight, and height (*p* > 0.05); however, there was a significant difference in the stiffness values (*p* < 0.05). An inverse correlation was observed between stiffness and the VAS score (*p* < 0.0001). A positive correlation was observed between the degree of curvature (*p* < 0.0001) and the time of curvature onset (*p* < 0.0001). The IIEF-15 scores were poorer in group A than in group B (*p* < 0.0001).

**Conclusion:**

SWE is an inexpensive, noninvasive method that can be used to measure the stiffness of PD patients.

## Résumé

**Contexte:**

Pour évaluer la rigidité de la tunique albuginée (TA), nous avons utilisé une nouvelle technique de diagnostic non invasive, appelée élastographie par ondes de cisaillement (EOC). Nous avons déterminé si les valeurs de EOC étaient corrélées avec le degré de courbure du pénis, le moment d’apparition de la maladie de Lapeyronie (MP) et la gravité de la douleur ressentie par les patients pendant l’érection. Cette étude a analysé l’élasticité de la TA des patients atteints de MP par rapport à celle d’un groupe témoin. Nous avons également recherché toute corrélation entre la rigidité des corps caverneux et le degré de courbure, le temps écoulé entre le diagnostic et l’apparition de la courbure, et la gravité de la douleur érectile. Il s’agit d’une étude cas-témoins prospective impliquant 100 hommes enrôlés de septembre 2020 à août 2021. Les participants ont été assignés au groupe A (cas, n = 50), qui comprenait des hommes atteints de MP, avec ou sans douleur, et présentant une courbure du pénis, ou au groupe B (témoins, n = 50), qui comprenait des patients en bonne santé âgés de plus de 18 ans qui venaient à la clinique d’urologie pour des raisons autres que la MP. Les antécédents médicaux ont été recueillis pour tous les patients qui ont également subi un examen objectif, une évaluation échographique en mode B et une EOC. Le questionnaire de l’échelle visuelle analogique (EVA) de l’Indice international de la fonction érectile (IIEF-15) a été administré à tous les participants.

**Résultats:**

Il n’y avait pas de différences significatives entre les groupes en ce qui concerne l’âge, le poids et la taille ; toutefois, il y avait une différence significative dans les valeurs de rigidité (p<0,05). Une corrélation inverse a été observée entre la rigidité et le score EVA (p<0,0001). Une corrélation positive a été observée entre le degré de courbure (p<0,0001) et le moment de l’apparition de la courbure (p<0,0001). Les scores IIEF-15 étaient plus faibles dans le groupe A que dans le groupe B (p<0,0001).

**Conclusions:**

L’élastographie par ondes de cisaillement (EOC) est une méthode peu coûteuse et non invasive qui peut être utilisée pour mesurer la rigidité des patients atteints de MP.

## Introduction

Peyronie’s disease (PD) is an acquired connective tissue disorder of the penis named in honor of Francois de la Peyronie, who reported a first case in 1743 [1]. PD is characterized by the formation of a fibrous scar in the tunica albuginea (TA) of the penis, resulting in penile curvature. This plaque is formed because of the altered production of the extracellular matrix caused by the upregulation of tissue inhibitors of matrix metalloproteinases and myofibroblast activity [2, 3]. The cause of PD is unknown; however, it has been established that repeated episodes of trauma cause microvascular injuries and the deposition of inelastic fibrin, collagen types I and III, and calcium [4]. Additionally, PD patients often exhibit other fibrotic changes, such as Dupuytren’s contracture, which could suggest a possible genetic predisposition [5].

PD occurs in 3.2% to 8.9% of the male population, and it typically presents at 55 to 60 years of age; however, it can occur at any age [6, 7]. Traditionally, men with PD experience pain during sexual intercourse, erectile dysfunction, and emotional and physical distress [8]. Furthermore, erectile dysfunction caused by pathological disease progression greatly affects the sexual activity of men and their partner [8].

Two phases of pathology can be distinguished. The first is the acute inflammatory phase during which plaque develops in the TA and begins to alter the anatomy of the penis. Pain is usually felt when the penis is in the flaccid state or during erection. The subsequent chronic phase involves the formation of a hard, palpable plaque and the stabilization of penile curvature [9]. Pain usually resolves in 90% of patients within 12 months of disease onset [10].

The diagnosis of PD is based on the medical history, sexual history, autophotography, clinical examination, and ultrasound imaging modalities. The diagnostic examination should begin with the detection of plaque; then, it should extend to the hands and feet to identify possible Dupuytren’s contracture. To perform a more complete evaluation, ultrasonography is the primary imaging technique used to detect plaques and identify calcification in lesions [11]. Additionally, penile Doppler ultrasound enables the identification of potential concomitant vascular compromise through the analysis of the penile vasculature and flow [12].

Shear wave sonoelastography (SWE) is a noninvasive technique that provides encouraging results for PD patients. It has been applied in other andrological fields, such as for the diagnosis of erectile dysfunction or for supporting the diagnosis of male infertility [13]. Additionally, SWE is a painless technique that quantitatively measures tissue elasticity by computing the modulus of elasticity (or Young’s modulus) expressed in kilopascals (kPa) or meters per second (m/s) and localizing the most rigid areas of the penis, such as penile plaques that are not visible using B-mode ultrasonography [14].

This study aimed to analyze the elasticity of the TA of PD patients compared to that of the control group and to determine any statistically significant differences. The secondary aim of this study was to analyze any correlations between the stiffness of the cavernous bodies and the degree of curvature, time to curvature onset, and erectile pain severity. Additionally, we tested whether there were significant differences in male sexual function using the International Index Erectile Function (IIEF-15) long-form questionnaire.

## Materials and methods

**Fig. 1:**
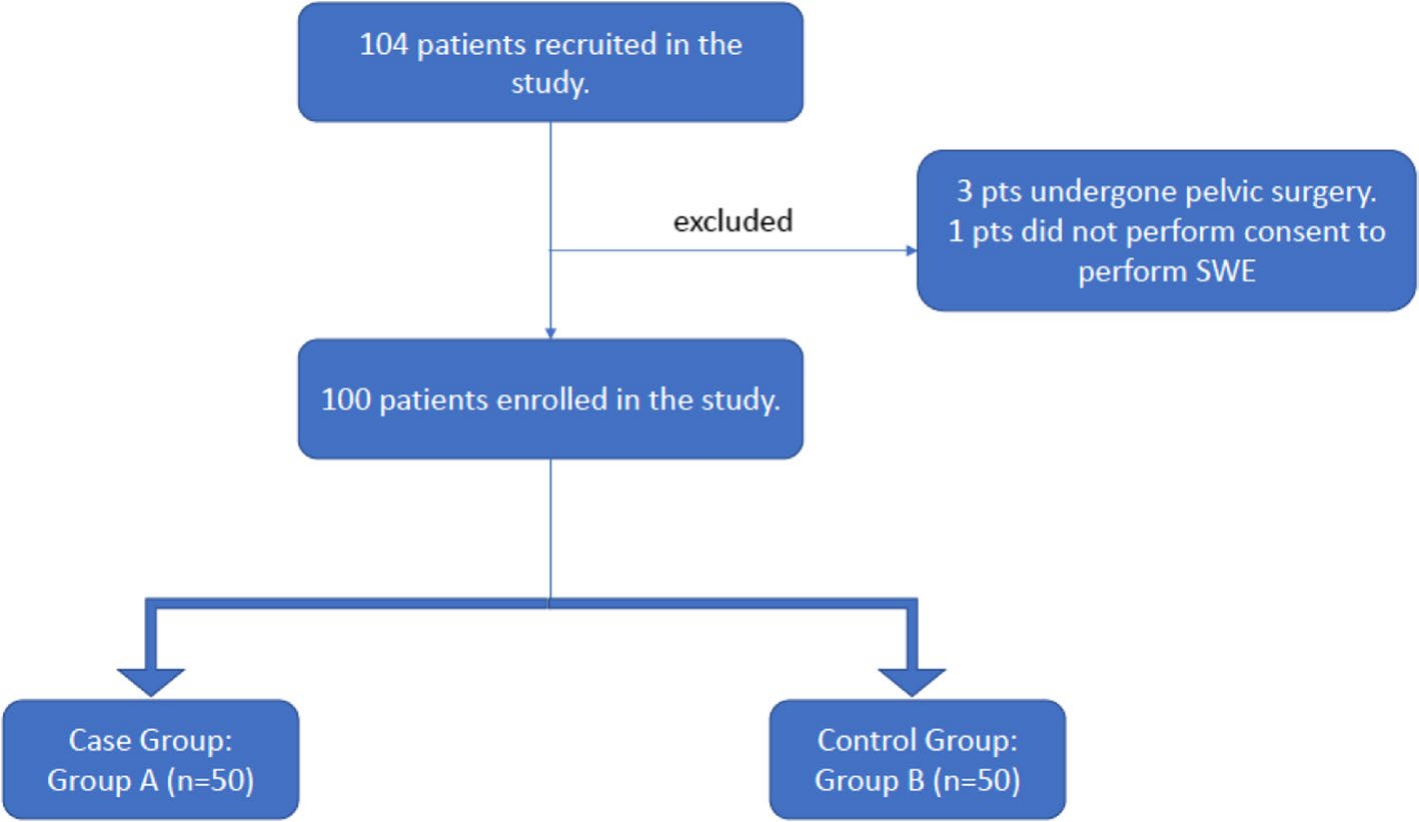
Flowchart of the study

**Fig. 2:**
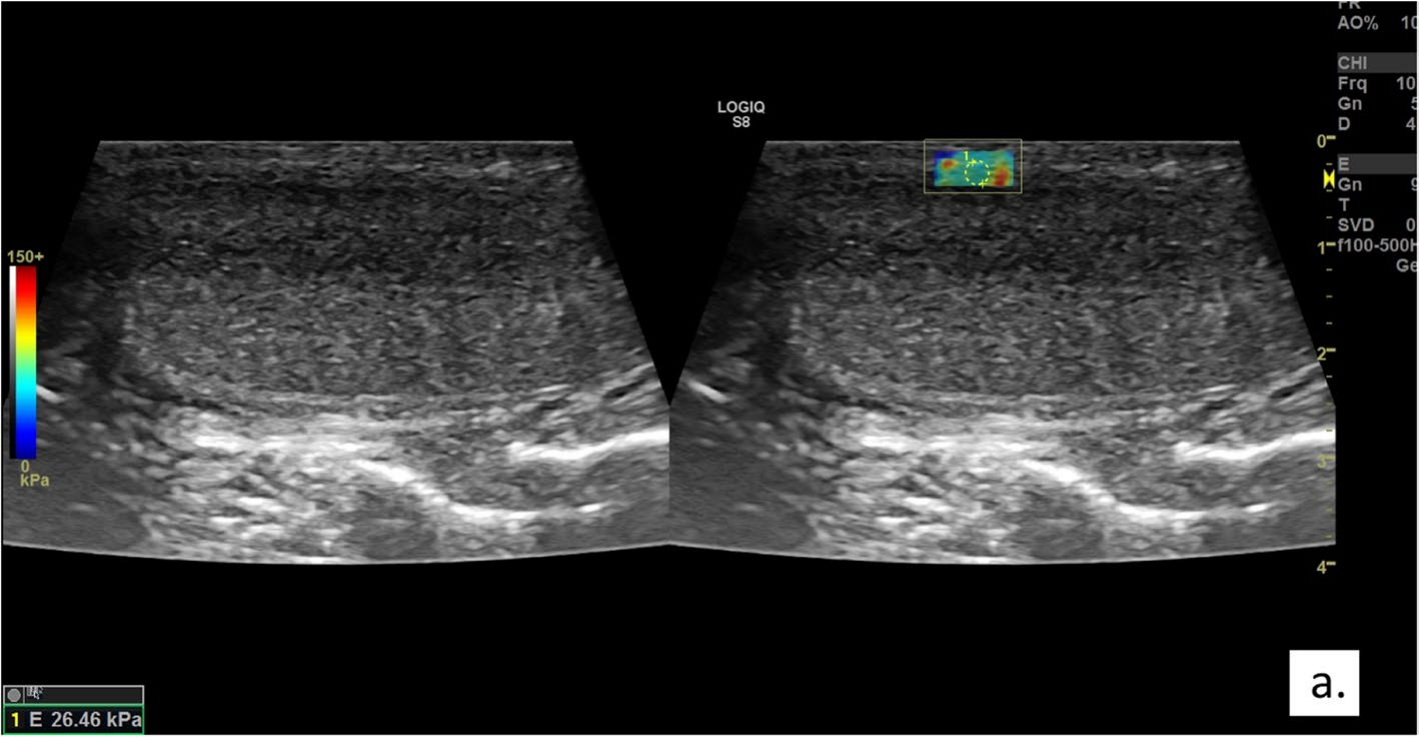
Example of a shear wave elastography image of Tunica Albuginea (TA) including quantitative measurement data expressed in kilopascal (kPa) in a patient with Peyronie's Disease (PD) (case group). The elastosonography mode simultaneously provides two images of the same area: The left image is in US—B—mode while on the right image is simultaneously displayed for stiffness measurement. The yellow box indicates the region of interest (ROI) observed in the shear wave elastography image, and the yellow circles indicate the intensity of the stiffness expressed values in kPa. In addition, the software provides a colorimetry map of the stiffness of the ROI. In particular, the red color indicates high stiffness while the blue color indicates lower stiffness. TA: tunica albuginea; kPa: kilopascal; PD: Peyronie’s Disease US: ultrasound; ROI: region of interest

This was a prospective case–control study. From September 2020 to August 2021, a total of 104 men at our urology clinic were considered eligible. However, four subjects were excluded (three had undergone pelvic surgery and one did not provide consent to undergo SWE). The participants were divided into two groups: group A (case group, *n* = 50), which included subjects older than age 18 years who were diagnosed with PD at any stage, with or without pain, and with penile curvature, and group B (control group, *n* = 50), which included healthy patients older than 18 years who visited the urology clinic for benign pathologies (Benign Prostatic Hyperplasia, urinary lithiasis, male infertility and IVU) excluding PD and neoplasia of the genitourinary system (Figs. 1 and 2). The exclusion criteria for both groups were as follows: diagnosis of PD; history of pharmacological or surgical treatments; previous pelvic or penile trauma; neurological diseases or peripheral neuropathy; treatment with phosphodiesterase-5 inhibitors within the last 3 months before the study; and use of a vacuum device as a recreational or therapeutic tool. Additionally, subjects with alterations in triglyceride, low-density lipoprotein cholesterol, and blood glucose levels were excluded.

The study protocol was reviewed and approved by the Bioethics Committee of University of Perugia(IRB N°54,895)Informed consent was confirmed by the Institutional Review Board. The study was conducted in accordance with the Privacy Act and the Declaration of Helsinki.

The clinical histories of all of patients were collected. All patients underwent a physical examination. SWE of the TA was performed using the Logiq Healthcare Sq8 ultrasound system (GE, Chicago, IL, USA); a single urology specialist with at least 3 years of experience conducting SWE performed the examinations. The obtained images were analyzed by a second urologist using the Picture Archive and Communications System to ensure that the previously collected measurements were accurate. SWE was performed in a separate quiet room so the participants would be relaxed. All patients were placed in the supine position with the glans facing the pubic symphysis. SWE was conducted with the penis in an erectile state by performing an intracavernous injection of alprostadil 10 mcg.

The TA of two corpora cavernosa (CC) were analyzed separately. The linear probe (7.5–13 MHz) was placed parallel to each CC. Each CC was divided into three sections longitudinally from the base of the crura of the penis to the glans to create the proximal, middle, and distal sections (all were 2 cm in length).

Subsequently, a region of interest with a radius of 0.5 cm was selected and positioned in the previously identified individual sections of the left CC and right CC corresponding to the TA. The TA was identifiable as a hypoechogenic band of measurable thickness bordered by two thin hyper-reflective interfaces. Additionally, we maintained a field of view slightly wider than the lesion so that surrounding tissues could be assessed. We kept the transducer in a direction perpendicular to the skin surface.

We kept the transducer stationary and asked the patients to not breathe during the measurements. We did not apply any precompression with the transducer. We allowed several seconds for the image to stabilize before performing the measurement. Additionally, areas with cysts or vascular structures in the organ were excluded from the measurements. Three serial measurements were performed and the arithmetic mean was determined. With the use of a software, the extent of stiffness of the selected area (expressed in kPa) was displayed. SWE results are based on the speed of the transverse wave that is generated in the direction of the ultrasonic beam; therefore, it is possible to estimate the modulus of elasticity (or Young’s modulus) [15].

A single operator at the clinic induced an erection in subjects with PD using alprostadil (10 mcg) and used a dedicated goniometer to measure the degree of penile curvature. Erectile pain was assessed using the visual analog scale (VAS) with scores ranging from 0 (no pain) to 10 (maximum perceived pain) [16].

All patients completed the IIEF-15 long-form questionnaire that analyzes the following aspects of male sexuality: erectile function; orgasmic function; sexual desire; intercourse satisfaction; and overall satisfaction [17].

### Statistical analysis

Statistical analyses were performed using the unpaired *t*-test and Levene’s test for continuous parametric variables and an analysis of variance to investigate any statistically significant differences in scale-level dependent variables and nominal-level variables with two or more categories. Pearson’s correlation test was used for quantitative variables. All calculations were performed using SPSS (version 22.0; IBM Corporation, Armonk, NY, USA). Statistical significance was set at *p* < 0.05.

## Results

There were no statistically significant differences regarding age, weight, and height between case and control group' (Table 1).

**Table 1:** Demographic characteristics of the subjects

	**Group A (*n* = 50)**	**Group B (*n* = 50)**	***p*-value**^**a**^
Age, (mean ± SD)	56.2 ± 12.8	52.1 ± 17.8	0.197
Weight, kg (mean ± SD)	78.4 ± 11.1	78.6 ± 10.8	0.928
Height, cm (mean ± SD)	174.6 ± 14.4	175.6 ± 13.7	0.745

Group A is the case group. Group B is the control group

*SD* standard deviation

^a^Determined using the t-test

Statistically significant differences were observed between the mean of the left TA of groups A and B (*p* < 0.0001) and between the mean of the right TA of groups A and B (*p* < 0.0001) (Table 2). Additionally, the averages of the results of the left and right TAs of groups A and B were calculated, and the differences were statistically significant.

**Table 2:** Mean values (kPa) of the TA of the left and right CC in the two groups

	**Group A (*n* = 50)**	**Group B (*n* = 50)**	***p*-value**^**a**^
TA of the left CC (mean ± SD) (kPa)	61 ± 19.7	22.8 ± 5.8	0.0001*
TA of the right CC (mean ± SD) (kPa)	53.2 ± 21.3	22.7 ± 5.6	0.0001*
Average TA of CC (mean ± SD) (kPa)	57.1 ± 20.5	22.7 ± 5.7	0.0001*

Group A is the case group. Group B is the control group

^a^Determined using the t-test (*p* < 0.05)

*SD* standard deviation, *TA* tunica albuginea, *CC* corpus cavernosum, *kPa* kilopascal

Moreover, in group A, 28 subjects presented the following characteristics: 53.5% (*n* = 15) had dorsal curvature; 17.8% (*n* = 5) had left lateral curvature; 14.2% (*n* = 4) had right lateral curvature; and 14.2% (*n* = 4) had ventral curvature. Furthermore, the mean curvature was 53.5 degrees (standard deviation [SD], ± 16.3 degrees). Subjects reported that the mean time from the diagnosis of the condition to curvature onset was 8.5 months (SD, ± 4.6). Subjects in group A had a mean VAS score of 4.7 (SD, ± 2.6).

Correlations were observed by analyzing the stiffness of the TA of the right CC and left CC. The time from diagnosis until curvature onset, curvature, and VAS scores are shown in Table 3. All correlations were statistically significant (*p* < 0.0001).

**Table 3:** Pearson’s correlation obtained from both CC of the TA and the degree of curvature, time from diagnosis to curvature onset, and VAS score

		General score	Curvature (degrees)	Time of onset of curvature (months)	VAS score
General score (kPa)	Pearson’s correlation	1	0.657	0.757	-0.714
	*p*-value		0.0001^*^	0.0001^*^	0.0001^*^
Curvature (degrees)	Pearson’s correlation	0.657	1	0.788	-0.747
	*p*-value	0.0001		0.0001^*^	0.0001^*^
Time of onset of curvature (months)	Pearson’s correlation	0.757	0.788	1	-0.873
	*p*-value	0.0001^*^	0.0001^*^		0.0001^*^
VAS score	Pearson’s correlation	-0.714	-0.747	-0.873	1
	*p*-value	0.0001^*^	0.0001^*^	0.0001^*^	

*CC* corpus cavernosum, *TA* tunica albuginea, *VAS* visual analogue scale

Group A is the case group. Group B is the control group

^*^Pearson’s correlation *p* < 0.05

Table 4 shows the scores of the various domains of the IIEF-15 questionnaire for both groups. In all the domains, statistically significant differences were observed between groups (*p* < 0.0001).

**Table 4:** IIEF-15 scores of group A (*n* = 50) and group B (*n* = 50)

		Mean (kPa)	SD (kPa)	*p*-value
IIEF erectile function	Group A	13.92	5.979	0.0001^*^
	Group B	27.42	2.749	
IIEF orgasmic Function	Group A	6.28	1.666	0.0001^*^
	Group B	8.26	1.440	
IIEF sexual desire	Group A	4.76	1.733	0.0001^*^
	Group B	8.46	1.446	
IIEF intercourse satisfaction	Group A	5.42	2.635	0.0001^*^
	Group B	13.34	2.096	
IIEF overall satisfaction	Group A	4.78	1.930	0.0001^*^
	Group B	8.00	1.604	

Group A is the case group. Group B is the control group

*IIEF* International Index of Erectile Function

^*^Determined using the t-test (*p* < 0.05 is statistically significant

## Discussion

PD is an insidious condition that has been underdiagnosed and underestimated by both patients and physicians for many years. Various diagnostic techniques have been used to diagnose PD [18].

Some radiological methods, such as radiography and computed tomography, have the ability to correctly visualize calcified plaques, but they are unable to properly investigate tissues without calcified plaques [19]. Magnetic resonance imaging is an appropriate technique for visualizing soft tissues and studying the CC and noncalcified plaques, and it can visualize areas of active inflammation usually present during the early phase of the disease [20]. However, it is less useful for visualizing calcific plaques and, more importantly, it is expensive and cannot be performed in an office [20].

The use of B-mode ultrasonography is currently the most preferred method in terms of cost and time, and it is advantageous because of its optimal visualization of calcified plaques. However, it fails to visualize noncalcified plaques located in complex areas such as the base of the penis. Moreover, it is unable to visualize areas of active inflammation and is operator-dependent. Furthermore, it is difficult to use as a follow-up method [21, 22]. Therefore, we are investigating new diagnostic techniques that are simple and easy to use. SWE results are based on the speed of propagation of sound waves generated by the probe through the tissues. Stiffer tissues with less elasticity will produce a higher speed of propagation of the wave, but more elastic tissues will produce a lower speed of propagation of the wave.

SWE has been applied in other areas. For example, it has been used in the field of endocrinology to study the thyroid and discriminate between benign nodules and malignant tumors [23], and in the field of breast cancer to distinguish between fibroadenomas and invasive tumors [24].

Additionally, the SWE technique, compared to other elastosonographic techniques, is operator-independent, is useful for monitoring patients after drug therapy, and can obtain values expressed in kPa.

Cavernous body biopsy is the gold standard for the study of histological changes in patients with PD. However, it may be infeasible because of ethical implications and the invasiveness of the procedure. Our study demonstrated that PD patients with elastic tissues of the CC and TA that were replaced by stiffer and less elastic tissues had statistically higher kPa values obtained by SWE than the control group.

Riversi et al. [25] showed that the use of a real-time elastography technique for PD patients allowed the detection of areas with less elasticity that were not visible with B-mode ultrasound. In fact, they analyzed 75 PD patients and observed that the combination of elastography and B-mode ultrasound was able to detect the lesion in 93% of subjects [25]. However, lesions were only detected in 86% of patients when using the B-mode technique and objective examination alone [25]. Moreover, similar to our data, SWE was able to identify lesions in the absence of palpable plaques. In fact, our results indicated that, regardless of the presence or absence of palpable plaques, PD patients had consistently higher stiffness values than the control group. The most plausible hypothesis is that the stiffness of the tissue increases with PD because of the abnormal production of the extracellular matrix, increased number of myofibroblasts, and production of collagen types I and III with fewer elastic fibers. Iacono et al. [26] reported that patients who undergo radical prostatectomy, which is associated with a PD incidence of approximately 15.9%, have decreased trabecular elastic fibers and smooth muscle fibers and significantly increased collagen content compared with patients who undergo preoperative biopsies [26]. Moreover, organized collagen and trabecular protocollagen deposits increased. This possibly occurs because of perioperative penile trauma and the release of cytokines that activate the abnormal wound healing process [5].

Hamidi et al. analyzed subjects who underwent radical prostatectomy with or without preservation of the nerve bundle [27]. They showed that patients with damaged nerve bundles had a statistically significant increase in penile stiffness and a consequent decrease in bundle length [27].

In PD patients, the normal structures of the TA and CC are essentially lost [12]. Specifically, it has been shown that there is an accumulation of myofibroblasts with subsequent activation and release of growth factors such as transforming growth factor-β1 and oxygen free radicals with the development of fibrosis and accumulation of collagen types I and III, thus leading to plaque formation over the long-term, curvature, and reduced penile length [27].

Moreover, Zhang et al. [28] reported that as stiffness measured by SWE increased, smooth muscle cells. Smooth muscle cells comprise a main component of the CC and approximately 50% of the penile microarchitecture; therefore, they have an important role in the erection process.

In another study, Zhang et al. [29] measured penile elasticity in erectile dysfunction patients and PD patients before and after a pharmacologically induced erection. They reported that there was a significant increase in viscoelasticity in patients with pharmacologically induced erections, demonstrating that SWE could be used to measure dynamic changes in erection [30].

Illiano et al. [30] analyzed 270 patients with various degrees of erectile dysfunction and observed a correlation between the worsening of IIEF-15 score and a higher degree of penile stiffness demonstrated by the erection hardness score; they also observed a reduction in elasticity demonstrated by an increase in kPa values diagnosed by SWE.

Qiao et al. [31] demonstrated an age-related increase in collagen fibers with an increase in penile stiffness. These data are in agreement with the epidemiology of PD, which has an incidence that increases with age.

SWE was able to detect an early increase in tissue stiffness, even in the absence of morphological features and detectable changes during objective examination, such as the presence of palpable plaque. Furthermore, it was found that as the VAS score increased, there was a negative correlation between the stiffness value of the TA, which concurs with the pathophysiology of the disease. During the acute phase of the disease, there are no structural changes in the TA and CC; these changes are usually present during the stable phase of PD, which is characterized by an increase in inelastic fibers compared to the quantity of elastic fibers.

Moreover, we found that with an increase in time from diagnosis to curvature onset and an increase in the degree of curvature, there was an increase in the stiffness (positive correlation) of the TA. This is because there is greater involvement of diseased tissues of the penis and diffuse fibrosis involving the total organ.

Our results are also in agreement with those of Illiano et al. [10], who studied men affected by PD who underwent surgery to plicate the TA. During that study, worsening of all areas of male sexual function (orgasmic, sexual desire, intercourse satisfaction, and overall satisfaction) was observed, but erectile function did not vary in a statistically significant manner preoperatively or postoperatively [10].

The limitations of this study include the small sample size and the failure to perform biopsies of the TA and the CC to correlate them with the kPa values obtained. Another limitation was that diagnostic confirmation with SWE was not performed after oral, local,or surgical therapy; therefore, any changes in kPa values could not be compared. In addition, the method is subject to inter/intra observer variability and therefore further studies are necessary to help Clinicians in standardizing the diagnostic procedure. The strengths of the study include the presence of a control group, the possibility of using stiffness as a reference to determine a possible conservative therapy response.

## Conclusions

SWE is a noninvasive and inexpensive ultrasound method that could be useful for evaluating PD patients. The measurement of TA stiffness in kPa could help monitor the pathology even after conservative therapies. Additionally, SWE could be used to diagnose PD earlier than B-mode ultrasonography alone. PD patients could be distinguished from the control group using the SWE and kPa values, which were correlated in a statistically significant manner with the degree of curvature, the time from diagnosis to curvature onset, and patient-perceived pain.

## Data Availability

Data are available on request because of privacy/ethical restrictions.

## Abbreviations

CC: Corpora cavernosa
IIEF-15: International Index of Erectile Function-15
kPa: Kilopascal
PD: Peyronie’s disease
SWE: Shear wave elastosonography
TA: Tunica albuginea
VAS: Visual analog scale

## References

[1] Akkus E, Levine LA (2007). Historical review of Peyronie’s disease. Peyronie’s Disease: a guide to clinical management.

[2] Del Carlo M, Cole AA, Levine LA (2008). Differential calcium independent regulation of matrix metalloproteinases and tissue inhibitors of matrix metalloproteinases by interleukin-1beta and transforming growth factor-beta in Peyronieonieki Diseasroblasts. J Urol.

[3] Ziegelmann MJ, Bajic P, Levine LA (2020). Peyronie's disease: Contemporary evaluation and management. Int J Urol.

[4] Gonzalez-Cadavid N, Rajfer J (2005). Mechanisms of disease: New insights into the cellular and molecular pathology of. Peyronie’s disease Nat Clin Pract Urol.

[5] Bias WB, Nyberg LM, Hochberg MC, Walsh PC (1982). Peyronie’s disease: a newly recognized autosomal-dominant trait. Am J Med Genet.

[6] Nehra A, Alterowitz R, Culkin DJ, Farady MM, Hakim LS, Heidelbaugh JJ (2015). Peyronie’s disease: AUA Guideline. J Urol.

[7] Capoccia E, Levine AL (2018). Contemporary review of Peyronie’s disease treatment. Curr Urol Rep.

[8] Illiano E, Trama F, Mancini V, Ruffo A, Romeo G, Riccardo F (2021). Peyronie’s disease may negatively impact the sexual experience of a couple and female sexual function: a single center study. Transl Androl Urol.

[9] Ralph D, Gonzalez-Cadavid N, Mirone V, Perovic S, Sohn M, Usta M (2010). The management of Peyronie’s disease: evidence-based 2010 guidelines. J Sex Med.

[10] Mulhall JP, Schiff J, Guhring P. An analysis of the natural history of Peyronie’s disease. J Urol 2006;175: 2115–8; discussion 2118. 10.1016/S0022-5347(06)00270-9 16697815

[11] Prando D (2009). New sonographic aspects of Peyronie’s disease. J Ultrasound Med.

[12] Madhumita P, Masterson JM, Masterson TA (2020). The role of imaging in the diagnosis and management of Peyronie’s disease. Curr Opin Urol.

[13] Illiano E, Trama F, Ruffo A, Romeo G, Riccardo F, Crocetto F (2021). Testicular shear wave elastography in oligo-astheno-teratozoospermic individuals: a prospective case-control study. Int Urol Nephrol.

[14] Richards G, Goldenberg E, Pek H, Gilbert BR (2014). Penile sonoelastography for the localization of a non-palpable, non-sonographically visualized lesion in a patient with penile curvature from Peyronie's disease. J Sex Med.

[15] Taljanovic MS, Gimber LH, Becker GW, Latt LD, Klauser AS, Melville DM (2017). Shear-Wave Elastography: Basic Physics and Musculoskeletal Applications. Radiographics.

[16] gould d, kelly d, goldstone l, gammon j. visual analogue scale (vas). j clin nursing 2001;10:697–706 10.1046/j.1365-2702.2001.00525.x 11822520

[17] Rosen RC, Riley A, Wagner G, Osterloh IH, Kirkpatrick J, Mishra A (1997). The international index of erectile function (IIEF): a multidimensional scale for assessment of erectile dysfunction. Urology.

[18] Parmar M, Masterson JM, Masterson TA (2020). The role of imaging in the diagnosis and management of Peyronie's disease. Curr Opin Urol.

[19] Andresen R, Wegner HE, Miller K, Banzer D. Imaging modalities in Peyronie's disease. An intrapersonal comparison of ultrasound sonography, X-ray in mammography technique, computerized tomography, and nuclear magnetic resonance in 20 patients. Eur Urol 1998;34:128–34. 10.1159/000019698 9693248

[20] Fornara P, Gerbershagen HP (2004). Ultrasound in patients affected with Peyronie's disease. World J Urol.

[21] Bertolotto M, Pavlica P, Serafini G, Quaia E, Zappetti R (2009). Painful penile induration: imaging findings and management. Radiographics.

[22] Kalokairinou K, Konstantinidis C, Domazou M, Kalogeropoulos T, Kosmidis P, Gekas A (2012). US imaging in Peyronie’s disease. J Clin Imaging Sci.

[23] Veyrieres JB, Albarel F, Lombard JV, Berbis J, Sebag F, Oliver C (2012). A threshold value in shear wave elastography to rule out malignant thyroid nodules: a reality?. Eur J Radiol.

[24] Cosgrove DO, Berg WA, Doré J, Skyba DM, Henry JP, Gay J (2012). Shear wave elastography for breast masses is highly reproducible. Eur Radiol.

[25] Riversi V, Tallis V, Trovatelli S, Belba A, Volterrani L, Iacoponi F (2012). Realtime-elastosonography of the penis in patients with Peyronie's disease. Arch Ital Urol Androl.

[26] Iacono F, Giannella R, Somma P, Manno G, Fusco F, Mirone V (2005). Histological alterations in cavernous tissue after radical prostatectomy. J Urol.

[27] Hamidi N, Altinbas NK, Gokce MI, Suer E, Yagci C, Baltaci S (2017). Preliminary results of a new tool to evaluate cavernous body fibrosis following radical prostatectomy: penile elastography. Andrology.

[28] Zhang JJ, Qiao XH, Gao F, Bai M, Li F, Du LF (2015). Smooth muscle cells of penis in the rat: noninvasive quantification with shear wave elastography. Biomed Res Int.

[29] Zhang X, Zhou B, Miranda AF, Trost LW (2018). A novel noninvasive ultrasound vibro-elastography technique for assessing patients with erectile dysfunction and Peyronie disease. Urology.

[30] Illiano E, Trama F, Ruffo A, Romeo G, Riccardo F, Iacono F (2021). Shear wave elastography as a new, non-invasive diagnostic modality for the diagnosis of penile elasticity: a prospective multicenter study. Ther Adv Urol.

[31] Qiao XH, Zhang JJ, Gao F, Li F, Liu Y, Xing LX (2017). An experimental study: evaluating the tissue structure of penis with 2D-ShearWave™ Elastography. Int J Impot Res.

